# Saffron Induced Relaxation in Isolated Rat Aorta via Endothelium Dependent and Independent Mechanisms

**Published:** 2018

**Authors:** Bibi Marjan Razavi, Ali Alyasin, Hossein Hosseinzadeh, Mohsen Imenshahidi

**Affiliations:** a *Department of Pharmacodynamics and Toxicology, School of Pharmacy, Mashhad University of Medical Sciences, Mashhad, Iran. *; b *Pharmaceutical Research Center, Pharmaceutical Technology Institute, Mashhad University of Medical Sciences, Mashhad, Iran.*

**Keywords:** *Crocus sativus* L, Saffron, Isolated rat aorta, L-NAME, Indomethacin, Hypertension

## Abstract

*Crocus sativus* L. (saffron) is a widely used food additive for its color and taste. The hypotensive effects of saffron have been shown in previous studies. The aim of this study was to evaluate the mechanism of vasodilatory effects induced by saffron on isolated rat aorta.

To study the vasodilatory effects of saffron aqueous extract (0.5, 1 and 2 mg/mL), isolated rat thoracic aorta rings were contracted by 10^−6^ M phenylephrine (PE) or KCl 80 mM. The vasodilatory effect of saffron was also evaluated both on intact and denuded endothelium aortic rings. To study the role of nitric oxide and prostacyclin in relaxation induced by saffron, aortic rings were incubated by L-NAME (10^-6^ M) and indomethacin (10^-5^ M) respectively for 20 min. Saffron induced relaxation in endothelium-intact aortic rings precontracted with PE in a concentration dependent manner. The obtained relaxation induced by the highest saffron concentration in endothelium-intact aortic rings precontracted with KCl was less than that observed in endothelium-intact aortic rings precontracted with PE. The relaxant activity of saffron was abolished by incubation of aortic rings with L-NAME but not in the presence of indomethacin. Also, the vasodilatory activity of saffron was partially abolished in endothelium denuded aortic rings. Saffron induced relaxation in isolated rat aortic rings might be due particularly to its effect on endothelium via nitric oxide synthase pathway and partly due to the effect on vascular smooth muscle cells via L type voltage dependent calcium channels.

## Introduction

*Crocus sativus* L. (saffron) is a perennial herb which belongs to the Iridaceae family and widely cultivated in Spain, Iran, and other countries (1). Saffron is an extensively used food additive for its color and taste and has been used in traditional as well as modern medicine to treat several illnesses (2). According to chemical analysis, more than 150 chemicals are present in saffron stigmas (3). Three main components of saffron which are responsible for its pharmacological effects including: crocins, the principle coloring agent (mono and diglycosyl esters of a polyene dicarboxylic acid, named crocetin) (4), the glycoside picrocrocin which is a precursor of safranal and responsible for its bitter taste and safranal, a monoterpen aldehyde which is the deglycosylated form of picrocrocin and is responsible for the characteristic aroma of saffron (5). In traditional medicine, saffron has been used because of several properties such as antispasmodic, eupeptic, anticatarrhal, nerve sedative, carminative, diaphoretic, expectorant, stimulant, stomachic, aphrodisiac, and abortion (2, 6). It is also believed that saffron can treat snoring, toothache, otitis, anal pain, and gout (2). Moreover, different pharmacological properties have been attributed to saffron and its active components including anticonvulsant (7, 8), antidepressant (9), anti-inflammatory (10-12) and antinociceptive (13, 14), antioxidant (15, 16) antitumor (17, 18) and hypotensive (19, 20) effects. In addition, saffron extract or its active constituents showed protective effects on some toxic agents including diazinon and acrylamide in rat brain, liver and cardiovascular system (21).

**Figure 1:**
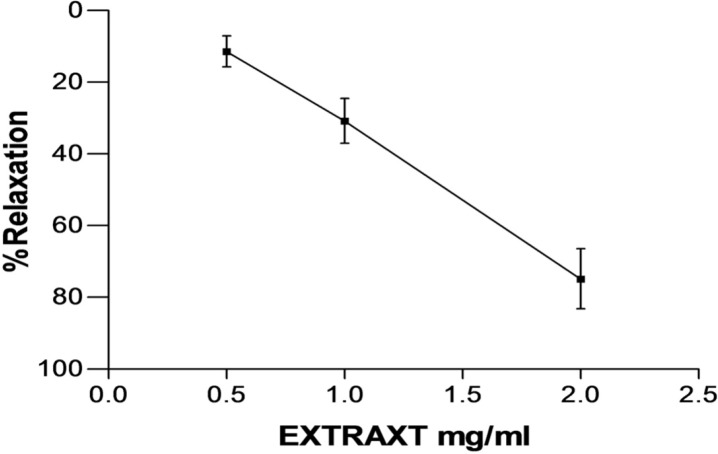
Effect of saffron aqueous extract in endothelium-intact aortic rings precontracted by PE (10-6 M(. The vasodilatory effect of saffron was expressed as a percentage of relaxation to maximum constriction induced by 10−6 M PE. Values are expressed as mean ± SEM. PE: phenylephrine.

**Figure 2:**
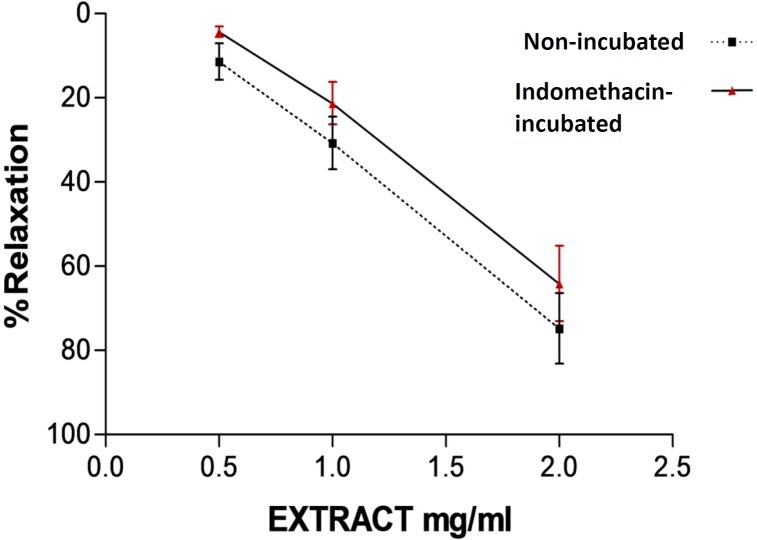
Relaxation effect of saffron aqueous extract in endothelium-intact aortic rings in non-incubated and incubated tissues with indomethacin (10^-5^ M) for 20 min. Then aortic rings were contracted by 10^−6^ M PHE and the vasorelaxant effect of saffron was then examined. Values are expressed as mean ± SEM. PE: phenylephrine.

**Figure 3:**
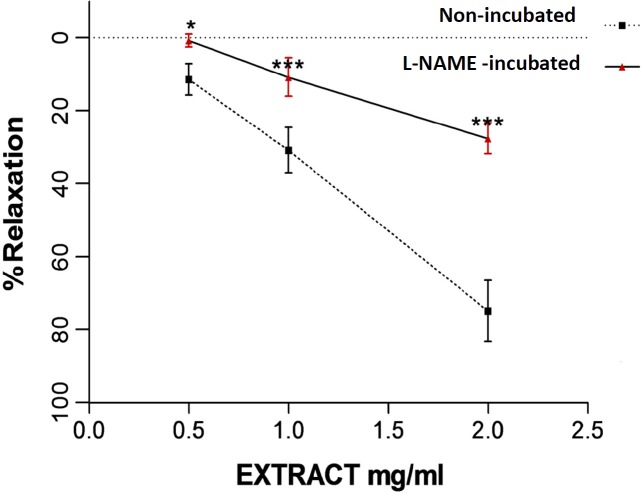
Relaxation effect of saffron aqueous extract in endothelium-intact aortic rings in non-incubated and incubated tissues with L-NAME (10-6 M) for 20 min. Then aortic rings were contracted by 10^−6^ M PE and the vasorelaxant effect of saffron was then examined. Values are expressed as mean ± SEM. ^*^P < 0.05 and ^***^P < 0.001 *vs. *L-NAME preincubated rings. PE: phenylephrine

**Figure 4:**
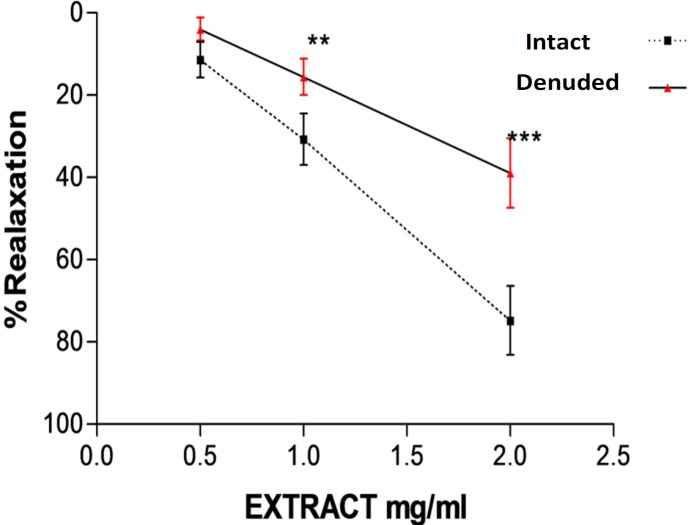
**. **Relaxation effect of saffron aqueous extract in endothelium denuded aortic rings precontracted by PE (10^−6^ M(. The vasodilatory effect of saffron was expressed as a percentage of relaxation to maximum constriction induced by 10^−6^ M PHE both on intact endothelium and denuded endothelium rings. Values are expressed as mean ± SEM. ^**^P < 0.01 and ^***^P < 0.001 *vs. *endothelium intact aortic rings. PE: phenylephrine.

**Figure 5:**
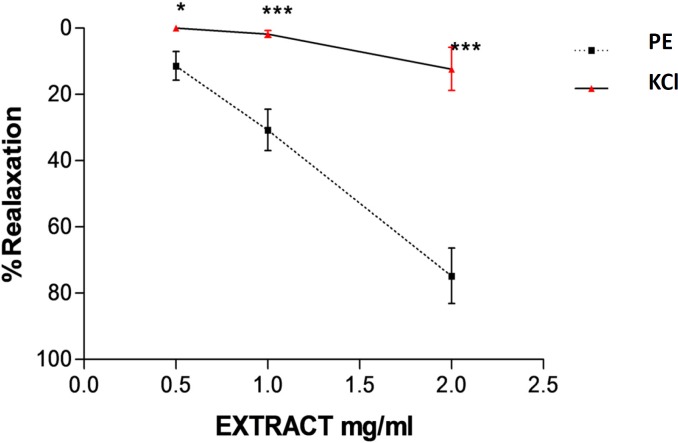
Effect of saffron aqueous extract in endothelium-intact aortic rings precontracted by KCl (80 m M(. The vasodilatory effect of saffron was expressed as a percentage of relaxation to maximum constriction induced by KCl. Values are expressed as mean ± SEM. ^*^P < 0.05 and ^***^P < 0.001 *vs. *PE precontracted rings, PE: phenylephrine.

Hypertension is one of the highly common cardiovascular diseases and considered as an important risk factor for developing other diseases such as metabolic syndrome, endothelial dysfunction, renal dysfunction, diabetes, congestive heart failure, coronary artery disease and stroke (22). Although many antihypertensive drugs are available, however, some adverse effects have been reported following antihypertensive drugs (23). Therefore, natural plants and their active components may be considered as new antihypertensive drugs with safety, efficacy, cultural acceptability and lesser side effects (24). 

The cardiovascular protective effects of saffron have been shown in several studies. The aqueous-ethanol extract of *C. sativus* could inhibit calcium channel of guinea-pig heart (25). In addition, saffron aqueous extract protected the heart against isoproterenol-induced myocardial infarction in Wistar rats (26). Moreover, the aqueous and ethanolic extracts of *C. sativus* petals reduced the blood pressure in a dose-dependent manner (27). The hypotensive activity of aqueous extract of saffron stigma has been shown in normotensive and hypertensive anaesthetized rats (19). In another study, chronic administration of saffron aqueous extract reduced the mean systolic blood pressure in desoxycorticosterone acetate (DOCA) salt treated rats (28). 

Considering the hypotensive effect of saffron, the aim of this study was to find out whether saffron shows vasodilatory effects on isolated rat aorta. In addition, the mechanism of vasodilatory effects induced by saffron on isolated rat aorta was evaluated.

## Experimental

*Plant extract*

*C. sativus* stigma from Novin Saffron Co. was collected from Ghaen, Khorasan province, Northeast of Iran. The plant was identified by Mazhari, and a voucher specimen (No. 11135), deposited at the Herbarium of Mashhad School of Pharmacy, was analyzed in accordance to the ISO/TS 3632, 1-2: 2003. The aqueous extract of *C. sativus* was prepared using maceration method. Briefly, 8 g of stigma powder was macerated in 300 mL distilled water for 72 h with continuous shaking in the refrigerator. After centrifuging, supernatant was freeze-dried for 24 h.

*Animal*

Adult male Wistar rats (weight 200–250 g) were provided by animal center (School of Pharmacy, Mashhad University of Medical Sciences). They were kept on a 12 h light/dark cycle and at a temperature of 23 ± 1 °C with free access to food and water. These conditions were maintained constant throughout the experiments. All animal experiments were carried out in accordance to Mashhad University of Medical Sciences, Ethical Committee Acts. 

*Drugs*

Phenylephrine hydrochloride (PE), acetylcholine chloride (ACh), indomethacin and N^G^-nitro-L-argininemethylester (L-NAME) were purchased from Sigma-aldrich (Germany). Other chemicals used in this study were provided from Merck (Germany).

*Tissue preparation*

Rats were killed by intraperitoneal (IP.) injection of ketamine/xylazine. Then the thorax was opened and the thoracic aorta was quickly removed and cleaned from adherent connective tissues and cut into rings (4-5 mm in length). Special care was taken to ensure that the endothelial was not damaged during tissue preparation. Two stainless steel stirrups were passed through the lumen of each ring. One stirrup was connected to an isometric force transducer (PowerLab, ADInstrument, Australia) to measure tension in the vessels. The rings were placed in a 25 mL organ chamber (Organ Bath, 4 channels, ADInstrument, Australia) containing Krebs solution gassed with 95% O_2_/5% CO_2_, and maintained at 37 °C. The composition of Krebs solution was as follows: 118.0 mM NaCl, 4.7 mM KCl, 1.2 mM NaH_2_PO_4_, 1.2 mM MgSO_4_, 25.0 mM NaHCO_3_, 11.1 mM glucose, and 2.5 mM CaCl_2_ (pH 7.4) (29). Rings were placed under a resting tension of 2 g (in preliminary studies determined to be optimum), and allowed to equilibrate for 1 h. During this equilibrium period the physiological salt solution was replaced every 15 min. The LabChart 7.3 (ADInstruments) software was used for this study. 

*Vasodilatory effects of saffron aqueous extract on the thoracic aorta rings*

To study the vasodilatory effects of saffron, isolated rat thoracic aorta rings were contracted by 10^−6^ M PE or KCl 80 mM in two separate experiments, when the vasoconstriction curves of rings reached the plateau phase of the maximum tension, saffron aqueous extract (0.5, 1 and 2 mg/mL) was added, and the tensions were recorded. The vasodilatory effect of saffron aqueous extract was expressed as a percentage of relaxation to maximum constriction induced by 10^−6^ M PE or KCl 80 mM. 

The vasodilatory effect of saffron aqueous extract on constriction induced by PE on rat thoracic aorta was also performed both on intact endothelium and denuded endothelium rings. The endothelial cells were removed by gently rubbing the aorta between fingers for approximately 30 sec. To confirm that the endothelial layer has been removed, we tested with a single dose of acetylcholine (10^-6^ M) after phenylephrine (10^-6^ M) in denuded vessels. The maximum relaxation induced by acetylcholine was less than 30%.

To examine whether the saffron induced vasorelaxation, is mediated by the NO, aortic rings were incubated by LNAME (10^-5^ M) for 20 min. Then thoracic aortic rings were contracted by 10^−6^ M PE and the vasorelaxant effect of saffron aqueous extract (0.5, 1 and 2 mg/mL) was then examined.

To examine the role of cyclooxygenase pathway in saffron mediated vasorelaxation, aortic rings were incubated by indomethacin (10^-5^ M) for 20 min. Then the thoracic aortic rings were contracted by 10^−6^ M PE and the vasorelaxant effect of saffron (0.5, 1 and 2 mg/mL) was then examined.

*Statistical analysis*

All results are expressed as mean ± SEM. ANOVA followed by Tukey–Kramer tests were performed to compare means. *P*-values less than 0.05 were considered as significant.

## Results

*Saffron induced vasorelaxation in endothelium-intact aortic rings precontracted by PE*

As shown in Figure 1, saffron aqueous extract (0.5, 1 and 2 mg/mL) induced relaxation in endothelium-intact aortic rings precontracted with PE (10^−6^ M) in a concentration dependent manner. Maximum relaxation response induced by the highest concentration of saffron (2 mg/mL) was about 75%.

*Vasodilatory effect of saffron in endothelium-intact aortic rings preincubated with Indomethacin*

As shown in Figure 2, there is no significant difference in vasorelaxant responses induced by saffron in endothelium-intact aortic rings precontracted with PE (10^−6^ M) in the absence or presence of indomethacin (10^-5^ M). 

*Vasodilatory effect of saffron in endothelium-intact aortic rings preincubated with L-NAME*

Incubation with L-NAME, for 20 min induced the significant differences in relaxation induced by saffron aqueous extract (0.5, 1 and 2 mg/mL) in endothelium-intact aortic rings precontracted with PE (10^−6^ M) in comparison with relaxation induced by saffron in the absence of L-NAME (*P* < 0.05 and *P* < 0.001, respectively) (Figure 3).

*Vasodilatory effect of saffron in endothelium denuded aortic rings*

Significant differences were observed between relaxation induced by saffron (1 and 2 mg/mL) in endothelium intact and endothelium denuded aortic rings (*P* < 0.01 and *P* < 0.001, respectively) (Figure 4).

*Saffron could not induce vasorelaxation in endothelium-intact aortic rings precontracted by KCl*

As shown in Figure 5, saffron (0.5, 1 and 2 mg/mL) could not induce relaxation in endothelium-intact aortic rings precontracted with KCl (80 mM) similar to that of obtained in aortic rings preincubated by PE. Maximum relaxation response induced by the highest concentration of saffron (2 mg/mL) was about 12.4%. There are significant differences between relaxation induced by saffron aqueous extract (0.5, 1 and 2 mg/mL in endothelium-intact aortic rings precontracted by KCl and relaxation induced by saffron in endothelium-intact aortic rings precontracted by PE (*P* < 0.05 and 

*P* < 0.001, respectively) (Figure 5).

## Discussion

Our results showed that saffron could induce vasorelaxation in isolated rat aortic rings. The vasorelaxant effect of saffron might be due particularly to its effect on endothelium via nitric oxide synthase pathway and partly due to the effect on vascular smooth muscle cells via L type voltage dependent calcium channels.

The hypotensive effects of saffron have been shown in several studies. Intravenous injection of saffron aqueous extract dose-dependently reduced the mean arterial blood pressure. According to the study, the injection of 10 mg/kg of the aqueous extract caused 60 ± 8.7 mmHg reductions in mean arterial blood pressure in normotensive and hypertensive anaesthetized rats. This hypotensive effect did not induce reflex tachycardia (19). Furthermore saffron extract showed a potent relaxant effect on guinea-pigs tracheal chains partly due to the presence of safranal (30). Another study revealed that chronic administration of saffron aqueous extract could reduce the mean systolic blood pressure in DOCA salt treated rats (28). Moreover, the aqueous and ethanolic extracts of *C. sativus* petals reduced the blood pressure in a dose-dependent manner (27). Regarding the lowering effect of saffron on blood pressure, this study was undertaken to evaluate the mechanism of saffron hypotensive effect using isolated rat aortic rings.

Our results indicated that saffron could induce vasorelaxation in endothelium intact aortic rings precontracted with PE. To investigate whether the relaxing effect of saffron depended on endothelium mediated mechanisms, endothelium derived vasodilator relaxing factors such as nitric oxide and prostacyclin were evaluated (31). Nitric oxide is an endothelium releasing factor which produces by eNOS (endothelial nitric oxide synthase) and induces vascular smooth muscle relaxation through the activation of guanylyl cyclase (32). NO has an essential role in the regulation of normal and pathological blood pressure (33). 

To assess the contribution of NO in relaxation induced by saffron, the aortic rings were incubated by L-NAME. L-NAME is an inhibitor of nitric oxide synthase. Inhibition of NO by L-NAME reduced the relaxation induced by saffron. Maximum relaxation responses induced by the highest concentration of saffron (2 mg/mL) in the absence and presence of L-NAME were 75% and 27.6%, respectively. So, NO is partially involved in saffron relaxant activity. 

Prostacyclin (PGI2) is another endothelium releasing factor that is produced by endothelial cyclooxygenase. Prostacyclin leads to elevation of cyclic AMP and finally reduces the availability of calcium and induces smooth muscle cell relaxation (31). To verify the involvement of prostacyclin in saffron induced vasorelaxation, another experiment was carried out in the presence of indomethacin. The results showed that relaxation produced by saffron was not modified by inhibition of cyclooxygenase. Therefore, saffron vasorelaxation is not mediated through cyclooxygenase pathway. Taken together, our findings demonstrated that relaxation by saffron is partly related to endothelium derived factors. In order to verify this hypothesis, another experiment was carried out on endothelium denuded aortic rings. The observed relaxant responses in intact and denuded endothelium were different at the moderate and the highest saffron concentrations. Maximum relaxation response induced by the highest concentration of saffron (2 mg/mL) in endothelium denuded aortic rings was 40%. So it can be concluded that relaxation effects of saffron could be endothelium dependent. In this study, the effect of saffron on rat isolated aortic rings precontracted by KCl was also evaluated. KCl depolarizes the cell membrane and increases intracellular calcium via voltage dependent calcium channels of smooth muscle cells and this process leads to contraction (34). Our results showed that significant differences between relaxation induced by saffron in endothelium-intact aortic rings precontracted by KCl and PE have been observed. Maximum relaxation response induced by the highest concentration of saffron (2 mg/mL) in endothelium-intact aortic rings precontracted by KCl was about 13% and in endothelium-intact aortic rings precontracted by PE was about 75% So, according to these results it is suggested that L type calcium channels might be also partially affected by saffron. Our results are in agreement with the findings of another studies that showed the aqueous-ethanolic extract of saffron inhibited guinea pigs smooth muscle cell contraction via calcium channel blocking (30, 35). Moreover in a previous study, chronic administration of saffron aqueous extract also reduced blood pressure in DOCA salt hypertensive rats probably due to the several mechanisms including inhibitory effect on smooth muscle cell, blocking of calcium channel or inhibition of sarcoplasmic reticulum Ca2+ release into cytosol, interaction with GABA (A)-benzodiazepine receptor complex and endothelial dysfunction (28). According to chemical analysis, more than 150 chemicals are present in saffron stigmas (3). Crocin and safranal are two main components of saffron which are responsible for its pharmacological effects (4). Crocins are water soluble compounds in saffron stigmas and safranal is the main volatile agent with low water solubility and is responsible for the characteristic aroma of saffron (36). The vasorelaxation effects of saffron are attributed to the presence of these constitutes in saffron stigma. Further studies are required to confirm whether the vasorelaxation effect of the saffron aqueous extract is due to these compounds (crocin or safranal) or due to the synergistic effects of several constituents. 

## Conclusion

In summary, the results of the present study indicated that saffron induced relaxation in isolated rat aortic rings might be due particularly to its effect on endothelium via nitric oxide synthase pathway and partly due to the effect on vascular smooth muscle cells via L type voltage dependent calcium channels.
